# Podoplanin Gene Disruption in Mice Promotes *in vivo* Neural Progenitor Cells Proliferation, Selectively Impairs Dentate Gyrus Synaptic Depression and Induces Anxiety-Like Behaviors

**DOI:** 10.3389/fncel.2019.00561

**Published:** 2020-01-15

**Authors:** Ana Cicvaric, Hannah M. Sachernegg, Tamara Stojanovic, Dörte Symmank, Tarik Smani, Thomas Moeslinger, Pavel Uhrin, Francisco J. Monje

**Affiliations:** ^1^Center for Physiology and Pharmacology, Department of Neurophysiology and Neuropharmacology, Medical University of Vienna, Vienna, Austria; ^2^Center for Physiology and Pharmacology, Institute for Physiology, Medical University of Vienna, Vienna, Austria; ^3^Department of Medical Physiology and Biophysics, Institute of Biomedicine of Seville (IBiS)/University of Seville/CIBERCV, Seville, Spain; ^4^Center for Physiology and Pharmacology, Department of Vascular Biology and Thrombosis Research, Medical University of Vienna, Vienna, Austria

**Keywords:** neurogenesis, podoplanin, LTD, anxiety-like behavior, NGF, cell proliferation, the hippocampus

## Abstract

Podoplanin (Pdpn), a brain-tumor-related glycoprotein identified in humans and animals, is endogenously expressed in several organs critical for life support such as kidney, lung, heart and brain. In the brain, Pdpn has been identified in proliferative nestin-positive adult neural progenitor cells and in neurons of the neurogenic hippocampal dentate gyrus (DG), a structure associated to anxiety, critical for learning and memory functions and severely damaged in people with Alzheimer’s Disease (AD). The *in vivo* role of Pdpn in adult neurogenesis and anxiety-like behavior remained however unexplored. Using mice with disrupted *Pdpn* gene as a model organism and applying combined behavioral, molecular biological and electrophysiological assays, we here show that the absence of Pdpn selectively impairs long-term synaptic depression in the neurogenic DG without affecting the CA3-Schaffer’s collateral-CA1 synapses. Pdpn deletion also enhanced the proliferative capacity of DG neural progenitor cells and diminished survival of differentiated neuronal cells *in vitro*. In addition, mice with podoplanin gene disruption showed increased anxiety-like behaviors in experimentally validated behavioral tests as compared to wild type littermate controls. Together, these findings broaden our knowledge on the molecular mechanisms influencing hippocampal synaptic plasticity and neurogenesis *in vivo* and reveal Pdpn as a novel molecular target for future studies addressing general anxiety disorder and synaptic depression-related memory dysfunctions.

## Introduction

Podoplanin (Pdpn), also known as AGGRUS; GP36; Gp38; GP40; HT1A-1; OTS8; PA2.26; T1A; TI1A; T1A2; T1A-2, is a small (~40 kDa) transmembrane cell-surface sialomucin-like glycoprotein present in humans and several other species, which has been implicated in severe pathophysiological processes in some of the organs where it is constitutively expressed (Rishi et al., 1995; Williams et al., 1996; Breiteneder-Geleff et al., 1997; Williams, 2003; Shibahara et al., 2006; Gittenberger-de Groot et al., 2007; Vanderbilt et al., 2008; Uhrin et al., 2010; Astarita et al., 2012; Kolar et al., 2015). In particular, Pdpn is expressed in the developmental and adult mammalian brain, where aberrant and exacerbated cellular Pdpn expression has been associated with the genesis and expansion of malignant brain tumors (Cortez et al., 2010; Astarita et al., 2012). Under physiological conditions, however, Pdpn is profusely expressed in the hippocampal dentate gyrus (DG; Kotani et al., 2003; Cicvaric et al., 2016), a region where neurogenesis takes place. The endogenous function of Pdpn in the adult DG and its involvement in *in vivo* neurogenesis remained however unexplored.

Adult neurogenesis is a form of plasticity comprising the formation of newly developed neurons that can be functionally integrated into pre-established synaptic circuits (Eriksson et al., 1998; Colucci-D’Amato et al., 2006; Costa et al., 2015; Baptista and Andrade, 2018). Few regions of the mammalian brain, including the hippocampal DG, contain dividing progenitor cells capable of giving rise to newly formed functional neurons (Liu and Martin, 2006; Hagg, 2009; Ming and Song, 2011; Walton, 2012; Dennis et al., 2016); indicative of a high degree of functional specificity. Newly generated neurons in the hippocampus play key roles in memory acquisition and maintenance (Anacker et al., 2015; Goncalves et al., 2016; Hollands et al., 2017; Toda et al., 2019). The mechanisms linking hippocampal neurogenesis to memory functions remain however poorly understood. Some of the neural plastic changes occurring during long-term potentiation (LTP) and long-term depression (LTD) are proposed as putative mechanisms participating in the *in vivo* formation of memories (Malenka and Bear, 2004; Sajikumar and Frey, 2004) and both LTP and LTD have been independently associated to neurogenesis (Staubli and Lynch, 1990; Jouvenceau et al., 2006; Saxe et al., 2006, 2007; Kemp and Manahan-Vaughan, 2007; Malleret et al., 2010). Alterations in hippocampal neurogenesis are additionally associated with psychiatric disorders including depression and anxiety (Abrous et al., 2005; Trejo et al., 2008; Llorens-Martin et al., 2010; Petrik et al., 2012; Nishijima et al., 2013; Toda et al., 2019) and to the onset and development of memory-related human brain neuropathologies, e.g., Alzheimer’s disease (AD; Chuang, 2010; Demars et al., 2010; Hong et al., 2010, 2011; Hollands et al., 2016; Lazarov and Hollands, 2016). However, the molecular elements linking neurogenesis to either LTP (Staubli and Lynch, 1990; Staubli et al., 1990; Tononi and Cirelli, 2006) or LTD (Zeng et al., 2001; Nakao et al., 2002; Malleret et al., 2010) and to memory dysfunctions and psychiatric disorders remain unclear.

Using a Pdpn knockout mouse line that was earlier utilized for studies on the function of Pdpn in the lymphatic vascular system (Uhrin et al., 2010), we previously reported that Pdpn gene disruption results in altered spatial reference memory and impaired synaptic strengthening specifically at the neurogenic DG (not at CA3-to-CA1 synapses), and further unveiled podoplanin as a promoter of neuritogenesis and synaptic activity (Cicvaric et al., 2016). The selective functional requirement of Pdpn to a specific sub-hippocampal region (the DG), for proper synaptic strengthening, suggested to us that Pdpn could be involved in additional DG-specific functions important for learning and memory. Here, we present experimental evidence unveiling a novel role for Pdpn in hippocampal neurogenesis, DG specific synaptic depression and mood-related behavior. We show that Pdpn disruption *in vivo* promotes neural progenitor cell proliferation, selectively impairs DG LTD and induces anxiety-like behaviors in mice. The identification of molecular elements concomitantly influencing neurogenesis, memory-related synaptic plasticity and mood behaviors is critical for a better understanding of the brain function in health and disease.

## Materials and Methods

### Animals

Male Pdpn knockout mice (Pdpn^−/−^) and their wild-type littermate mice (Pdpn^+/+^), 9–18 weeks old, in 129S/v: Swiss background were obtained by crossing of heterozygous mice and maintained in specific pathogen-free facilities of the Medical University of Vienna. Animals were housed in groups of 3–5 mice per cage in a temperature [(22 ± 1)°C] and light [(200 ± 20)lx] controlled colony room with food and water provided *ad libitum*. The illumination was kept on a 12 h light/dark cycle with the light period starting at 6:00 a.m. Experiments described in this study were approved by the national ethical committee on animal care and use (BMWFW-66.009/0201-WF/II/3b/2014, Bundesministerium für Wissenschaft, Forschung und Wirtschaft), and carried out according to the U.K. Animals Scientific Procedures Act, 1986 and associated guidelines, EU Directive 2010/63/EU for animal experiments and in compliance with ARRIVE guidelines.

### Neurogenesis Analysis *in vivo*

#### BrdU Injections

**Figure 1:**
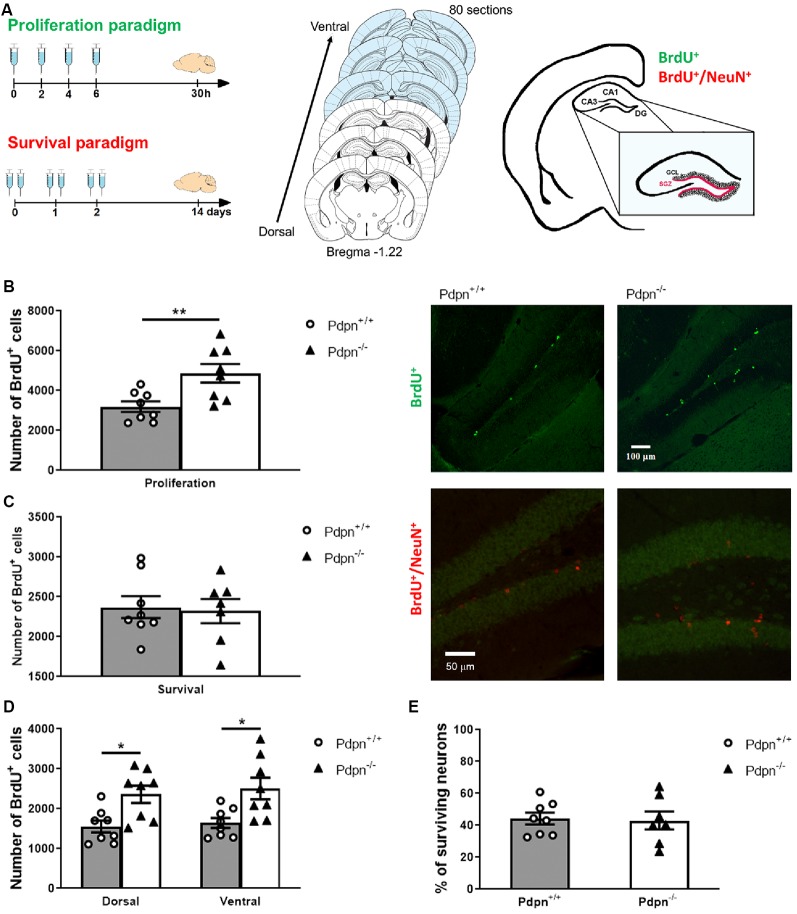
Lack of Podoplanin increases the proliferation of cells in the subgranular zone (SZG) of the dentate gyrus (DG). **(A)** Schematic representation of 5-bromodeoxyuridine (BrdU) injection protocols for proliferation and survival paradigms (left panel). Schematic representation span of the rostrocaudal axes from which coronal sections were collected (middle). The depicted coronal sections have been modified from Franklin and Paxinos (2013). The SGZ spans at the border of the granule cell layer (GCL) and hilus of the hippocampal DG (right panel). **(B)** Analysis of the approximated number of BrdU^+^ cells per hippocampus in “proliferation” paradigm showed a significant difference between two genotypes (**P = 0.008, *t*_(14)_ = 3.122, *n* = 8 per group). Representative photomicrographs of Pdpn^+/+^ and Pdpn^−/−^ coronal sections immunostained against BrdU in the “proliferation” paradigm (right panel 10× magnification). **(C)** In the “survival” paradigm quantification of BrdU^+^ cells showed no differences between hippocampi of Pdpn^+/+^ and Pdpn^−/−^ mice (*P* = 0.819, *t*_(13)_ = 0.233, *n* = 7–8 per group). Representative photomicrographs of Pdpn^+/+^ and Pdpn^−/−^ coronal sections immunostained against BrdU in red and against NeuN in green in the “survival” paradigm (right panel 20× magnification). **(D)** Two-way ANOVA of the number of BrdU^+^ cells in “proliferation” paradigm between dorsal and ventral hippocampus showed a main significant effect of genotype (***P < 0.0002, *F*_(1,28)_ = 17.61, *n* = 8 per group), no significant effect of the area (*P* = 0.5601, *F*_(1,28)_ = 0.3477, *n* = 8 per group) and no significant interaction between paradigm and genotype (*P* = 0.8912, *F*_(1,28)_ = 0.0190, *n* = 8 per group). *Post hoc* analysis showed significant difference between Dorsal:Pdpn^+/+^ and Dorsal:Pdpn^−/−^ (*P = 0.0366), Dorsal:Pdpn^+/+^ and Ventral:Pdpn^−/−^ (*P = 0.0108), as well as Ventral:Pdpn^+/+^ and Ventral:Pdpn^−/−^ (*P = 0.0233), as represented with asterisks on the graph. **(E)** Quantification of surviving cells double-labeled with BrdU^+^ and NeuN^+^ showed no significant difference in a number of surviving neurons in Pdpn^−/−^ mice compared to their Pdpn^+/+^ littermates (*t*_(13)_ = 0.1857, *P* = 0.9, *n* = 7–8). Data are displayed as mean ± SEM.

In order to analyze different stages of neurogenesis two injection paradigms were used following previously published protocols (Meshi et al., 2006; Pollak et al., 2008; Khan et al., 2014). Briefly, naïve mice were i.p. injected with BrdU [(+)-5′ Bromo-2′-deoxyuridine, Cat. No. B5002, Sigma–Aldrich, Vienna, Austria] dissolved in 0.9% NaCl solution at 50 mg/kg, four times (every 2 h) and sacrificed 24 h after the last BrdU injection to assess proliferation of progenitor cells (“proliferation paradigm”). For the assessment of the survival of the newborn cells (“survival paradigm”), mice were injected with the same dosage of BrdU twice per day (every 8 h) for three consecutive days and sacrificed 14 days after the first injection (as shown in Figure 1A).

#### Tissue Preparation

Upon induction of deep anesthesia *via* i.p. administration of a ketamine/xylazine cocktail (100 mg/kg ketamine, Ketanest^®^S, Pfizer Corporation Austria Gesellschaft m.b.H., Vienna, Austria and 40 mg/kg xylazine, Rompun^®^, Bayer, Germany), transcardial *in situ* perfusions were performed using 4% paraformaldehyde in phosphate-buffered saline (PBS, Cat. No. 70011-036, Thermo Fisher Scientific, Vienna, Austria). Brains were carefully extracted and stored in the fixative solution for 24 h and then transferred to 30% w/w sucrose in PBS solution for an additional 24 h. Serial sections of 30 μm thickness were cut using a cryostat (CM1950, Leica, Vienna, Austria) and stored in cryoprotective solution (30% ethylene glycol, 30% glycerol in PBS) at −20°C until used for immunolabeling. Eighty sections per animal were collected, starting at Bregma −1.22 where the hippocampal structure becomes visible (see Figure 1A, middle panel; Franklin and Paxinos, 2013).

#### Pretreatment for BrdU Immunohistochemistry

In order to visualize the BrdU incorporation into the nuclear DNA, it is necessary to perform the heat/acid DNA denaturation step (Boulanger et al., 2016). Additionally, and in order to protect the integrity of the tissue from the harsh heat/acid treatment, sections were first incubated in 50% formamide in 2× SSC buffer solution (Cat. No. 15557-044, 0.3 M NaCl, 0.03 M sodium citrate, Thermo Fisher Scientific, Vienna, Austria) at 65°C for 90 min, then rinsed in 2× SSC buffer solution, that was followed by incubation in 2 M HCl for 30 min at 37°C, and finally quick 10 min incubation in 0.1 M borate buffer (pH = 9).

#### Immunohistochemistry

Every 10th free-foating section of the entire rostrocaudal extent of the hippocampus was used for immunohistochemical analysis (*n* = 7–8 animals per group). Multicolor immunofluorescence staining protocols for mature granular neurons were applied basically as described elsewhere (Pollak et al., 2008; Khan et al., 2014). In essence, after multiple washing steps, sections were blocked with 10% normal donkey serum (NDS, Cat. No. 017-000-121, Jackson ImmunoResearch) in Trizma base solution (TBS, Trizma^®^ base, Sigma–Aldrich, Vienna, Austria) for 1 h, and subsequently incubated for 72 h at 4°C in 5% NDS blocking solution with following primary antibodies: anti-BrdU (marker of newly generated cells, 1:300, Cat. No. OBT0030G, Abd Serotec, Biorad, Vienna, Austria), anti-NeuN (mature neuron marker, 1:1,000, Cat. No. MAB377, Merck Millipore, Vienna, Austria). Next, sections were washed and incubated with appropriate secondary antibodies (1:500, Alexa Fluor^®^, Abcam, Cambridge, UK). Negative controls were performed by omitting the primary antibody. For the proliferation paradigm, cells were labeled with the anti-BrdU primary antibody and an Alexa Fluor secondary antibody conjugated with a 488 nm fluorophore. For the survival paradigm, sections were double-labeled with the anti-BrdU primary antibody and the Alexa Fluor secondary antibody conjugated with a 594 nm fluorophore and anti-NeuN primary antibody followed by an Alexa Fluor secondary antibody with a conjugated 488 nm fluorophore.

#### Stereology

Hippocampal immunofluorescence labeling was quantified conferring to a modified unbiased stereology protocol by an experimenter blinded to the genotype of the animals (Gould et al., 1999; Pollak et al., 2008). In the proliferation paradigm, the total number of cells in the well-defined region of the subgranular zone (SGZ) of the DG of both hippocampi in a 30 μm section was quantified throughout parallel series of sections (see Figure 1A, right). For the quantification of the number of cells, eight sections were quantified per animal. The resulting average number of cells per section per hippocampus was multiplied by the total number of sections to estimate the total number of newly generated cells. In the survival paradigm, all BrdU^+^ cells within the SGZ and approximately width of one cell above the SGZ, in order to account for cell migration were quantified in the same way as in the proliferation paradigm. The total number of BrdU^+^ cells in mice sacrificed 24 h after the last BrdU injection was used to evaluate the rate of proliferation. To assess survival rate, average numbers of BrdU^+^ cells per granule cell layer (GCL) per section were quantified in mice that were sacrificed 14 days following the BrdU injection. Quantification of the total number of cells that showed colocalization of BrdU labeling and immunoreactivity for mature granular neuronal marker- neuron-specific nuclear protein (NeuN^+^) was used to calculate the number of surviving neurons (Kempermann et al., 1997a,b; Khan et al., 2014). For double-labeled cells, only when it was confirmed that both Alexa Fluor 488 and Alexa Fluor 594 signals are in the same optical plane of focus, cells were scored as double-labeled. Cells that we in outer most, optical plane, were not scored in order to avoid counting cell caps (Huang et al., 1998). Fluorescent signals were detected using a Carl-Zeiss Axiovert-Apotome System (Oberkochen, Germany) coupled to Axiovision software version 4.8. at 20× magnification as previously described (Gould et al., 1999; Yu et al., 2009; Khan et al., 2014).

### Electrophysiology

#### Hippocampal Slices Preparation

Acute hippocampal slices were prepared from behaviorally naïve Pdpn^+/+^ and Pdpn^−/−^ male mice (*n* = 8–11 slices per group) following previously published protocols (Cicvaric et al., 2016, 2018a,b). In a nutshell, following a quick decapitation, animal’s brain was extracted into a cold artificial cerebrospinal fluid (aCSF) solution that had following composition (in mM): 125 NaCl, 2.5 KCl, 25 NaHCO_3_, 2 CaCl_2_, 1 MgCl_2_, 25 D-glucose, 1.25 NaH_2_PO_4_ with pH adjusted to 7.4. Next, transverse hippocampal slices (400 μm thick) were cut using a McIlwain Tissue Chopper (Mickle Laboratory Engineering, Guildford, Surrey, UK) and moved to recover for at least 1 h in the homemade submerged holding chamber that was maintained at (28 ± 2)°C. All solutions were perfused with carbogen gas mixture (95% O_2_, 5% CO_2_) during the course of the experiment.

#### Extracellular Recordings

For recording, individual slices were transferred to a submerged recording chamber of low volume that was perfused with a constant flow (2–3 ml/min) of carbonated aCSF solution. Evoked field excitatory postsynaptic potentials (fEPSPs) were recorded using a borosilicate glass pipettes that were pulled in a horizontal puller (Sutter Instrument, Novato, CA, USA) thus yielding a tip with resistance of 2–5 MΩ when filled with aCSF solution. Stimulation was done *via* custom-made Teflon-coated tungsten wire bipolar stimulating electrode (~50 μm diameter tip). The stimulating electrode and recording microelectrode tips were positioned to target two major functional regions of the hippocampal trisynaptic circuit, CA3-Schaffer Collateral-CA1 and medial perforant pathway (MPP) of DG following previously published protocols (Massa et al., 2011; Monje et al., 2012; Cicvaric et al., 2016, 2018a,b). Stimuli needed to produce 40–50% of maximal amplitude that was established by repeated stimulation with pulses of voltage (0–6 V, 1 V increments, 100 μs duration) were used for baseline recordings (pulse duration = 100 μs, 0.03 Hz). To induce LTD, low-frequency stimulation (LFS, 900 pulses at 1 Hz) protocol was delivered at baseline stimulation intensity using an ISO-STIM 01D isolator stimulator (NPI Electronics, Tamm, Germany). Synaptic depression was determined by analyzing the changes in the decaying phase of fEPSP slopes normalized to baseline, averaged within a group and compared between Pdpn^+/+^ and Pdpn^−/−^ mice. AxoClamp-2B amplifier (Bridge mode) and a Digidata-1440 interface (Axon Instruments, Molecular Devices, Berkshire, UK) were used for recordings and pClamp-10 software (Molecular Devices, Berkshire, UK) was used for offline analysis.

### Behavioral Testing

All experiments shown in this study were performed during the light phase of the light/dark cycle by an experimenter blinded to the genotype of the animals. Animals were singly housed for at least 1 week before the start of the experiment and handled daily by the experimenter for 5 min in order to reduce stress that is induced by manipulation of animals throughout the experiments. Additionally, on the day of each test, mice were transferred to their home cages for habituation to the testing room, 1 h prior to experiments. During habituation and throughout the experiments white noise generator was used so as to mask possible intermittent loud noises from the surroundings (Can et al., 2012).

#### Open Field Test

In addition to the evaluation of the alternations in general locomotor activity, the Open Field test (OFT) is used as a screening test for anxiety-related behavior (Prut and Belzung, 2003). Following previously published protocols (Cicvaric et al., 2018a,b), at the beginning of each session, Pdpn^+/+^ or Pdpn^−/−^ mouse was placed in the center of an Open Field arena (Cat No. ENV-510, Med Associates Inc., St. Albans, VT, USA) that consisted of four transparent acrylic glass walls (height 20.3 cm) on a white polyvinyl chloride plastic square board (side 27.3 cm), and allowed to explore the arena for 5 min. At the end of each session, the subject was placed back to the home cage and the apparatus was thoroughly cleaned with 70% ethanol and used again only once the odor volatilized. To test for any possible changes in locomotor activity and exploratory behavior distance that each animal traveled in the arena was recorded and analyzed using associated software, Activity Monitor (Cat No. SOF-812, Med Associates Inc., St. Albans, VT, USA). Likewise, to test for alterations in anxiety-like behavior, the time spent in the virtual center of the arena (~25% of the total area) and the number of entries to the center were analyzed (Bailey and Crawley, 2009).

#### Light Dark Box

To further test for specific influence that genetic-targeting of Pdpn could have on anxiety-like behavior in mice we firstly employed the Light/Dark Box test (LDB) essentially as described before (Leach et al., 2013; Cicvaric et al., 2018a). Briefly, the OFT arena was modified by inserting a specialized, opaque Dark Box Insert (Cat No. ENV-511, Med Associates Inc., St. Albans, VT, USA) to enclose two equal-sized compartments, connected *via* small passage, with one section illuminated and the other one darkened. At the start of the experiment, the animal was positioned in the bright section facing away from the passage and allowed to explore freely both sections for 10 min. Once the session was completed, the animal was returned to the home cage and the test arena was carefully whipped with 70% ethanol and used again only once the scent volatilized to prevent a bias based on olfactory cues. Since mice instinctively avoid the brightly lit compartment, percentage of time spent in the lit area as well as number of crossings between compartments were automatically measured and recorded by a specialized software package (Cat No. SOF-812, Activity Monitor, Med Associates, St. Albans, VT, USA) and used as experimental indices of anxiety.

### Elevated Plus-Maze

The elevated plus maze (EPM), currently one of the most popular tests to examine anxiety-like behaviors, is based on the mice’ innate fear of open, elevated areas. When given a choice, mice show an inclination to stay in the safe (closed arms) compared to risky (open arms) surroundings (Pinheiro et al., 2007; Ramos, 2008). Here, EPM was carried out following established, previously published protocols (Shumyatsky et al., 2002; Glangetas et al., 2017; Cicvaric et al., 2018a). Experiments were performed using a plus-shaped Plexiglas elevated (~50 cm above ground) apparatus (Viewpoint, Lyon, France) that consisted of two opposing closed and two opposing open arms. All arms of the EPM were 40 cm long and fitted with white floors, while closed arms had an additional dark insert with 20 cm high walls. Open arms were brightly illuminated (110 lx), while the closed arm had a dim illumination (15 lx). At the beginning of each 5 min session, the subject was placed at the juncture area (central square) so as to face one of the open arms and allowed to explore the maze without restrictions. Animal behavior was recorded and analyzed using an accompanying video tracking software (VideoTrack, Viewpoint, Lyon, France). Several conventional and ethological parameters like amount of time animal spent in the open arms, number of entries to open arms and total distance traveled in the maze during the test were evaluated as described before (Walf and Frye, 2007).

### Neurogenesis Analysis *in vitro*

#### Embryonic Mouse Neural Stem Cell Culture

Timed-pregnant Pdpn^−/−^ or Pdpn^+/+^ mice were killed by cervical dislocation and the pups quickly removed and decapitated in sterile Hank’s Balanced Salt Solution (HBSS, Cat. No. 14185052, GIBCO^®^, Thermo Fisher Scientific, Waltham, MA, USA) on Embryonic Day 15. Hippocampi were dissected, placed in a small volume of ice-cold standard Dulbecco’s Modified Eagle’s Medium (DMEM Thermo Fisher Scientific, Waltham, MA, USA) and used to prepare embryonic mouse neural stem cell (NSC) culture following standardized protocols described before (Brewer and Torricelli, 2007; Azari et al., 2011a,b,c; Siebzehnrubl et al., 2011). Dissected hippocampal tissue was cut and enzymatically dissociated using 1× Trypsin (Cat. No. 15090046, Thermo Fisher Scientific, Waltham, MA, USA) 37°C for 10 min. Trypsin was removed and dissociated tissue was gently washed four times with warm DMEM. Following mechanical dissociation, the cell suspension was centrifuged at 1,800 rpm for 2 min (Universal 320 from Hettich GMB, Tuttlingen, Germany, 10 cm rotor ratio, type 1554). Pellet was then re-suspended in warm Neurocult^TM^ basal medium (Cat. No. 05700, Stem Cell Technologies, Vancouver, BC, Canada) supplemented with Neurocult^TM^ proliferation supplement (Cat. No. 05701, Stem Cell Technologies, Vancouver, BC, Canada) and 20 ng/ml EGF (Cat. No. 02633, Stem Cell Technologies, Vancouver, BC, Canada) and plated at a density of 8 × 10^4^ viable cells/cm^2^. Neurospheres were harvested at 5 DIV and frozen in 10% DMSO in Neurocult^TM^ basal medium.

#### Differentiation

Cells were cultured following standard procedures but never sub-cultured (passaged) more than once in order to avoid possible alterations in self-renewal capability due to many passages (Azari et al., 2011c). After quick thawing frozen aliquots of the NSC were centrifuged and the pellet re-suspended in warm Neurocult^TM^ basal medium supplemented with Neurocult^TM^ proliferation supplement and 20 ng/ml EGF. After 24 h, primary spheres were passaged by incubation in 0.1× Trypsin for 1.5 min at 37°C. The activity of Trypsin was stopped with Trypsin inhibitor (Cat. No17075029). Thermo Fisher Scientific and the spheres were then mechanically dissociated into a single-cell suspension. 0.2 × 10^6^ viable cells per coverslip for both genotypes were plated in DMEM medium (Cat. No. 21103049, Thermo Fisher Scientific, Waltham, MA, USA) supplemented with B27 supplement (Cat. No. 17504044, Thermo Fisher Scientific, Waltham, MA, USA). Coverslips were double-coated, first with Poly-L-Lysine (Cat. No. P6282, Sigma–Aldrich, Vienna, Austria), followed by a coat of laminin (Cat. No. L2020, Sigma–Aldrich, Vienna, Austria) to allow cells to adhere and differentiate. Cells were treated continuously every 24 h with NGF (50 ng/ml in DMEM with B27) for 4 or 8 days.

#### Immunocytochemistry

Cells were fixed with 4% PFA in PBS solution at appropriate time points. First, cells were blocked with 5% normal goat serum (NGS, Cat. No. X0907, Dako) and 5% normal donkey serum (NDS, Cat. No. 017-000-121, Jackson ImmunoResearch, West Grove, PA, USA) in PBS and then further incubated with anti-glial fibrillary acidic protein (GFAP; Cat. No. SAB5500113, rabbit polyclonal, 1:500, Sigma–Aldrich, St. Louis, MO, USA), anti-microtubule associated protein 2 (MAP2; Cat. No. ab5392, chicken polyclonal, 1:25,000, Abcam, Cambridge, UK) primary antibodies that were diluted in PBS containing 0.1% BSA (A8022, Sigma–Aldrich, St. Louis, MO, USA), 1% NGS, 1% NDS and 0.1% Triton X-100 (Cat. No. T8787, Sigma–Aldrich, St. Louis, MO, USA) for 48 h at 4°C. Next, cells were washed with PBS and subsequently incubated with Alexa Fluor 488 conjugated goat-anti-chicken IgGs (Cat. No. A11039, 1:1,000, Thermo Fisher Scientific, Waltham, MA, USA) and Alexa Fluor 594 conjugated donkey-anti-rabbit IgGs (Cat. No. A21207, 1:1,000, Thermo Fisher Scientific, Waltham, MA, USA) secondary antibodies diluted in 2% BSA in PBS solution for 1 h at room temperature. Negative controls were produced by omitting primary antibodies. Cell nuclei were stained with 4′,6-diamidino-2-phenylindole (DAPI, Cat. No. D1306, 1:1,000, Thermo Fisher Scientific, Waltham, MA, USA) diluted in PBS for 5 min at room temperature. Cells were washed with PBS, mounted with Fluoro-Gel mounting medium (Cat. No. 17985–10, Electron Microscopy Sciences, Hatfield, PA, USA) and viewed with a conventional fluorescence microscope. Six non-overlapping images per coverslip were captured at 40× magnification with a Zeiss Axiovert Colibri7 microscope and exported using the ZEN 2.3 (blue edition) software (Zeiss, Jena, Germany). Only cells overlapping with DAPI were further manually counted with ImageJ (NIH) using a cell counter plugin (Kurt De Vos, ImageJ, NIH).

### Statistical Analysis

Statistical analyses were done using GraphPad Prism Software, version 7.0 (San Diego, CA, USA). To test for the normality of data, the D’Agostino’s K^2^ test was used prior to further statistical analyses. Two-sided unpaired student’s *t*-test was used for two-group comparisons and two-way ANOVA followed by Tukey’s test to account for multiple comparisons unless otherwise indicated. *P*, *t*, and *F* values together with the number of samples used (n) are presented in the figure legends. All data are expressed as means ± standard error. *P* < 0.05 was considered significant. *P < 0.05, **P < 0.01, ***P < 0.001, ****P < 0.0001. All source data used for the preparation of graphs and statistical analysis and other relevant data that support the conclusions presented in the study are available from the authors upon request.

## Results

### Lack of Pdpn Increases Proliferation of Cells in the Subgranular Zone of the Dentate Gyrus

To study the involvement of Pdpn in adult neurogenesis, we used a previously characterized Pdpn knockout mice (Uhrin et al., 2010; Cicvaric et al., 2016) and sought out to analyze in these mice the properties of cell proliferation and survival in the SGZ of the hippocampal DG by using BrdU-labeling as a strategy to determine newly formed cells (Figure 1A). Quantitative analysis 24 h post-injection indicated a significant increase in the number of BrdU^+^ cells in the SGZ of the Pdpn^−/−^ compared to Pdpn^+/+^ mice (Figure 1B). No statistically significant differences in the number of BrdU^+^ cells were observed between SGZs of Pdpn^−/−^ and Pdpn^+/+^ mice 14 days post-injection, indicating that while lack of Pdpn augmented the proliferation of neural progenitor cells in the SGZ, there was no difference in the number of surviving cells 2 weeks after BrdU injection (Figures 1B,C). Considering previously shown the differential contribution of the dorsal and ventral hippocampus to different sets of behaviors, we sought out to determine whether the changes we see in the proliferation rates will be confined to one and not the other part of the hippocampus. We found that while differences in rates of the proliferation between genotypes were conserved there was no significant difference in the proliferation rates between the dorsal and ventral hippocampus (Figure 1D). To determine whether the perturbations in proliferation could have had influence specifically on the survival of mature neurons, we performed multiplex labeling experiments with the neuron-specific nuclear protein NeuN and BrdU (BrdU^+^/NeuN^+^). No significant difference in the percentage of surviving neurons between Pdpn^−/−^ and Pdpn^+/+^ mice was observed (Figure 1E).

### Pdpn Deletion Alters Long-Term Depression in the Hippocampal Dentate Gyrus

**Figure 2:**
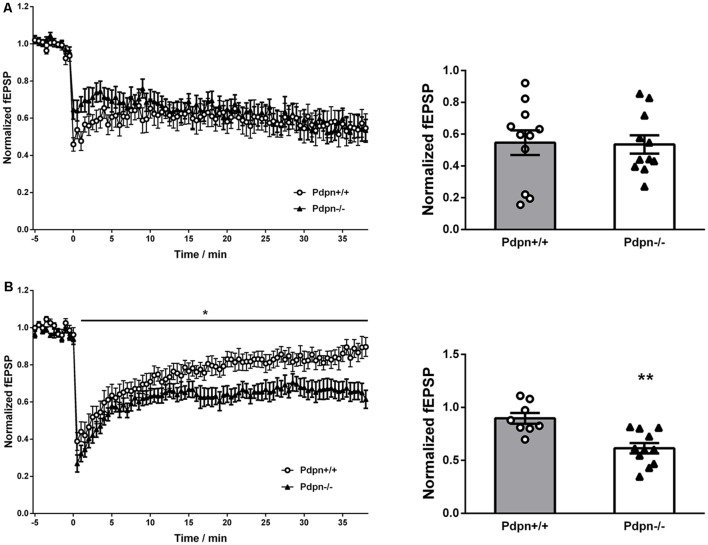
Podoplanin deletion alters long-term depression (LTD) selectively in the hippocampal DG. **(A)** Temporal courses of averaged slopes of fEPSP in CA3-Schaffer collateral-CA1 synapses (left panel) showed a strong main significant effect of time (****P < 0.0001, *F*_(76,1520)_ = 3.72, *n* = 11), no significant main effect of genotype (*P* = 0.6, *F*_(1,20)_ = 0.36, *n* = 11) and a significant interaction between time and genotype (***P = 0.0004, *F*_(76,1520)_ = 1.7). Corresponding bar graphs of the end-time points (right panel) showed no significant differences between Pdpn^+/+^ and Pdpn^−/−^ (*t*_(20)_ = 0.1124, *P* = 0.9, *n* = 11). (**B**) Temporal courses of averaged slopes of fEPSP in medial perforant pathway (MPP) of DG (left panel) showed a strong main significant effect of time (****P < 0.0001, *F*_(75,1275)_ = 27.87, *n* = 8–11), a moderate significant main effect of genotype (*P = 0.03, *F*_(1,17)_ = 5.99, *n* = 8–11) and a significant interaction between time and genotype (****P < 0.0001, *F*_(75,1275)_ = 1.88). Corresponding bar graphs of the end-time points (right panel) showed significant differences between Pdpn^+/+^ and Pdpn^−/−^ (*t*_(17)_ = 3.93, **P = 0.002, *n* = 8–11). Data are displayed as mean ± SEM.

In order to examine if Pdpn could be involved in bidirectional synaptic plasticity, we applied protocols of low-frequency electrical stimulation (LFS) to both the CA3-to-CA1 and MPP synaptic pathways in acute hippocampal slices from Pdpn^+/+^ and Pdpn^−/−^ mice. Data showed that the induction and maintenance of LTD in CA3-Schaffer Collateral-CA1 pathway synapses of Pdpn^−/−^ mice were indistinguishable from those from wild-type animals (Figure 2A). However, when LFS protocol was applied to the MPP pathway, it resulted in stronger maintenance of the induced LTD in slices from Pdpn^−/−^ mice as compared to those from their control wild type counterparts (Figure 2B).

### Effects of Pdpn Deletion on Anxiety-Like Behavior

**Figure 3:**
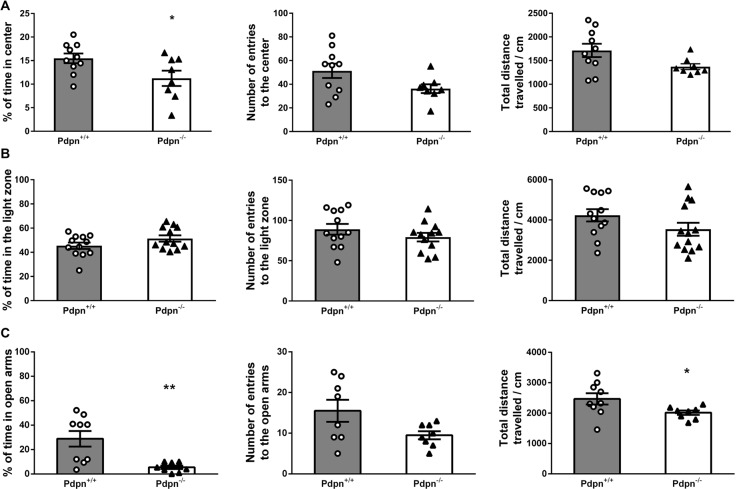
Effects of podoplanin deletion on anxiety-like behavior. Anxiety-like behavior was evaluated using several tests. **(A)** In Open Field test (OFT) significant difference in percentage of time spent in the brightly lit center between Pdpn^−/−^ and their wild-type littermate was found (*t*_(16)_ = 2.29, *P = 0.04, *n* = 8–10). No significant differences were observed in total distance traveled and the number of entries to the brightly lit center (*t*_(16)_ = 2.05, *P* = 0.06, *n* = 8–10 and *t*_(16)_ = 1.99, *P* = 0.07, *n* = 8–10). **(B)** No differences were observed in the percentage of time spent in the light area of the Light/Dark Box test (LDB) arena (*t*_(22)_ = 1.61, *P* = 0.2, *n* = 12). Mice from both groups also showed comparable values for the total distances traveled and the number of entries to the LDB arena (*t*_(23)_ = 1.54, *P* = 0.2, *n* = 12–13 and *t*_(22)_ = 1.16, *P* = 0.3, *n* = 12). **(C)** Pdpn^−/−^ animals presented with significantly less time spent in the open arms of the elevated plus-maze (*t*_(15)_ = 3.37, **P = 0.005, *n* = 8–9) and significantly less traveled distance in the open arms (*t*_(15)_ = 2.16, *P = 0.05, *n* = 8–9), as compared to their wild type littermates. No significant differences between groups were observed in the number of entries to the open arms (*t*_(14)_ = 2.08, *P* = 0.06, *n* = 8). Data are displayed as mean ± SEM.

Since the process of neurogenesis has been previously linked to the expression of mood-related behaviors (Revest et al., 2009; Parihar et al., 2011; Anacker et al., 2018), we next implemented widely standardized behavioral tests aiming to examine whether absence of Pdpn would result in possible changes in anxiety-like behaviors. We first examined Pdpn^+/+^ and Pdpn^−/−^ mice in the OFT and observed that Pdpn^−/−^ mice showed significantly higher anxiety-like levels compared to their wild type littermates as indicated by a reduction in percentage of the time that Pdpn^−/−^ mice spent in the center of the Open field arena (Figure 3A, left panel). No differences were found, however, in total distance traveled or the number of entries to the brightly lit center, indicating an absence of locomotor defects resulting from Pdpn deficiency (Figure 3A, middle and right panel). When both groups of animals were examined in the LDB, data indicated no significant differences in the percentage of time mice spent in the light area of the LDB arena, total distance traveled or number of entries to the light area of the test arena (Figure 3B). In the Elevated Plus Maze test (EPM), a very significant reduction in the time Pdpn^−/−^ mice spent in the open arms of the maze was apparent, indicating increased levels of anxiety in Pdpn^−/−^ mice compared to their wild type littermates (Figure 3C, left panel). A reduction in the distance traveled during the test in the open arms was also observed (Figure 3C, middle panel). No significant differences between groups were observed in the number of entries to the open arms (Figure 3C, right panel).

### Lack of Pdpn Selectively Decreases the Survival of Neuronal Cells That Is Rescued by NGF Treatment

**Figure 4:**
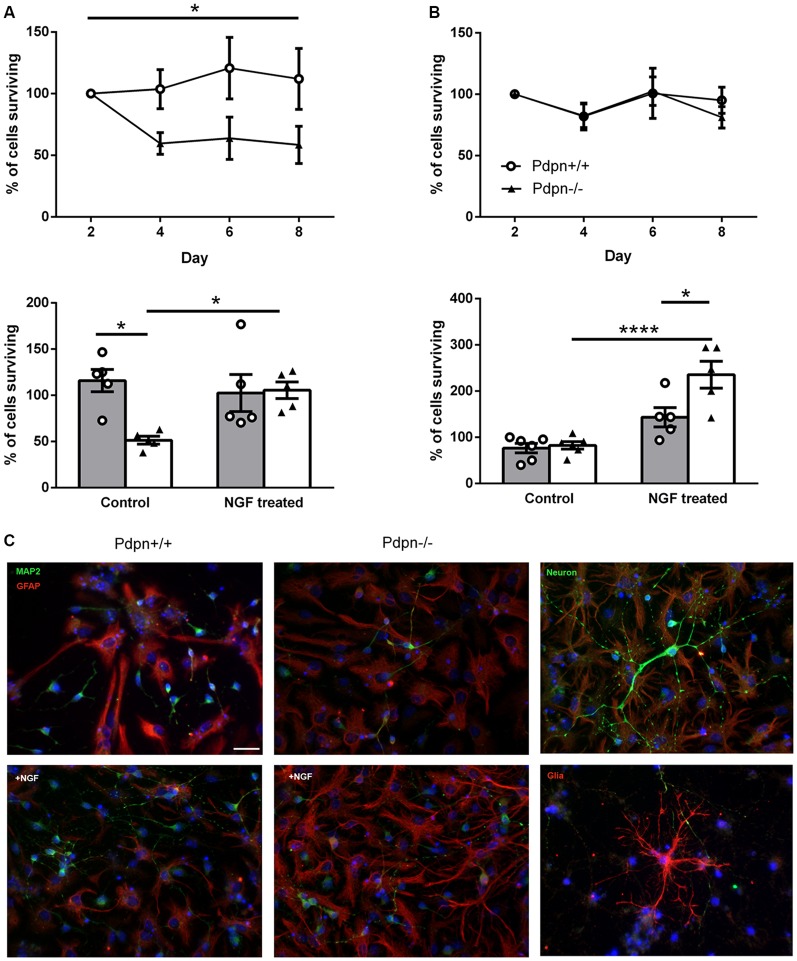
Lack of Podoplanin selectively decreases the survival of neuronal cells that are rescued by NGF treatment. Percentage of **(A)** neuronal and **(B)** glial cells derived from Pdpn^+/+^ and Pdpn^−/−^ neurospheres surviving during 8 days (upper panels), showed a significant main effect of the genotype on number of surviving neurons (*P = 0.02, *F*_(1,10)_ = 8.69, *n* = 6) but not on the number of glial cells (*P* = 0.8, *F*_(1,8)_ = 0.07, *n* = 5). No significant effects of time were observed on a number of neuronal or glial cells. Percentage of **(A)** neuronal and **(B)** glial cells derived from Pdpn^+/+^ and Pdpn^−/−^ neurospheres surviving after 4 days of NGF treatment, lower panels. In neuronal cells two-way ANOVA showed no significant effect of treatment (*P* = 0.2, *F*_(1,16)_ = 2.556, *n* = 6) but a significant effect of genotype (*P = 0.03, *F*_(1,16)_ = 5.84, *n* = 6) and a significant interaction between treatment and genotype (*P = 0.02, *F*_(1,16)_ = 7.07). *Post hoc* analysis showed a significant difference between Pdpn^+/+^ and Pdpn^−/−^ without treatment (*P = 0.02) and between Pdpn^−/−^ with and without NGF treatment (*P = 0.059), as represented with asterisk on the graph. Analysis of glial cells showed a strong main significant effect of treatment (****P < 0.0001, *F*_(1,18)_ = 38.73, *n* = 5), significant main effect of the genotype (*P = 0.02, *F*_(1,18)_ = 7.695, *n* = 5) and a significant interaction between two factors (*P = 0.03, *F*_(1,18)_ = 5.942, *n* = 5). *Post hoc* analysis showed a significant difference between Pdpn^−/−^ with and without NGF treatment (****P < 0.0001) and between Pdpn^+/+^ and Pdpn^−/−^ when treated with NGF (*P = 0.02), as represented with an asterisk on the graph. **(C)** Representative images of differentiated neurospheres immunolabeled with neuronal (MAP2) and glial (GFAP) markers (left and central pictures). In the right, upper and lower pictures, comprise representative images of immuno-based identified neurons (upper) and glial (lower) cells. Data are displayed as mean ± SEM.

On the basis of our observations from *in vivo* neurogenesis experiments in Pdpn^−/−^ mice, indicating significantly increased proliferation in the SGZ without alterations in the number of surviving neurons even after 2 weeks of cell-labeling, we asked ourselves whether this transitory enhanced proliferation phenotype was followed by cell death of newly-generated neuronal cells. In order to explore this possibility using an entirely different experimental approach, we prepared neurosphere cultures from wild type and Pdpn^−/−^ mice, as other groups have successfully proven the advantages of conducting *in vitro* assays using NSCs when studying the structural morphological as well as the functional signaling mechanisms underlying cell differentiation and neurogenesis (Schramm and Schulte, 2014). Following cell differentiation, we tracked the survival of neuronal and glial cells throughout 8 days at 2 days intervals. The phenotype of the cells was determined using the specific markers microtubule-associated protein 2 (MAP2) for neurons and GFAP for glial cells (Figure 4C). While there were no significant differences in the survival of glial cells from Pdpn^+/+^ and Pdpn^−/−^ mice, there was a significant sharp decrease in the number of surviving neuronal cells in cultures derived from Pdpn^−/−^ mice, starting at day 4 (Figures 4A,B, upper panels). A particular strength of this assay its ability to easily study the activity of soluble factors on NSCs. We have previously shown that Pdpn can physically interact with NGF (Cicvaric et al., 2016), a very functionally relevant neurotrophin known to hold a significant role in the differentiation and survival of new neurons (Levi-Montalcini, 1987a,b). We therefore next quantified the number of neuronal and glial cells on day 4 following differentiation in the presence or absence of NGF. In neuronal cells, NGF treatment significantly increased the percentage of Pdpn^−/−^ surviving cells, thus bringing it to the levels comparable to the Pdpn^+/+^ cells with or without NGF treatment, suggesting that NGF rescued the effect of Pdpn deletion. In glial cells, NGF treatment increased the number of Pdpn^−/−^ surviving cells.

## Discussion

### Pdpn and Neurogenesis in the Adult Brain

The role of Pdpn has been widely studied in last decades in several different disciplines including vascular physiology, cardiology, urology, and pulmonology). In the brain, Pdpn has been also shown to be expressed in hippocampal neurons and in nestin-positive neural progenitor stem cells in the SGZ of the hippocampal DG (Kotani et al., 2002, 2003; Astarita et al., 2012; Tomooka et al., 2013; Song et al., 2014), a region central for the acquisition of memory functions (Goncalves et al., 2016; Milner and Klein, 2016). Yet, most of the available studies about Pdpn in the brain have focused on its involvement in the promotion of brain tumors (Astarita et al., 2012; Peterziel et al., 2012; Grau et al., 2015; Kolar et al., 2015) and the intrinsic role of Pdpn in neurogenesis and memory-related synaptic plasticity in the adult brain remained undetermined. Here, we propose Pdpn as a molecular player involved in the functional fine-tuning of hippocampal neurogenesis. In agreement with our results here, previous reports had linked Pdpn to the regulation of cellular proliferation (Ramirez et al., 2003; Williams, 2003; Spinella et al., 2009; Cortez et al., 2010; Yan et al., 2011; Peterziel et al., 2012; Acton et al., 2014; Grau et al., 2015); and novel *in vitro* models studying stem cell and glioma invasion have also revealed elevated levels of Pdpn in migrating and invasive NSCs (Sailer et al., 2013). Additionally, studies in P19 embryonic carcinoma cells that can be differentiated into neuronal cells, and in neuronal stem cells that were sorted out based on their Pdpn expression levels, had shown that precursor cells with higher levels of Pdpn expression exhibit higher self-renewal capabilities and greater variability in their differentiation profiles (multi-lineage differentiation into neural cells), whereas precursor cells with lower levels of Pdpn expression displayed low self-renewability and differentiated predominantly into neurons (Kotani et al., 2002, 2003, 2007). Studies* in vitro* from other groups had moreover shown the presence of Pdpn in hippocampal nestin-positive neural progenitor cells (Kotani et al., 2002, 2003, 2007). All these observations are in agreement with our findings and support our hypothesis for an involvement of Pdpn in the regulation of the *in vivo* proliferative properties of hippocampal neural progenitor stem cells; our *in vivo* neurogenesis experiments show that deletion of Pdpn significantly increases the proliferation of NSCs in the SZG of the DG, hence hinting that Pdpn might be part of a proliferation suppression pathway (Figure 1B).

The findings linking Pdpn to hippocampal neurogenesis are timely and relevant. Neurogenesis has been described in several species and is associated to the genesis of a number of severe brain neuropathologies (Jin et al., 2004; He and Shen, 2009; Chuang, 2010; Demars et al., 2010; Spalding et al., 2013; Regensburger et al., 2014; Bergmann et al., 2015; Ernst and Frisén, 2015; Verdaguer et al., 2015; Frisén, 2016; Kang et al., 2016; Boldrini et al., 2018; Kempermann et al., 2018; Lucassen et al., 2019; Moreno-Jiménez et al., 2019). Neurogenesis is also implicated in the regulation of synaptic plasticity and learning and memory processes (Snyder et al., 2001; Dupret et al., 2008; Akers et al., 2014; Lazarov and Hollands, 2016). However, the mechanism regulating neurogenesis, synaptic plasticity and mood- and memory-related functions continue to be poorly understood and the specific signaling pathways linking Pdpn to these processes remain to be elucidated. There are several molecular and functional interacting partners of Pdpn that are expressed in brain neurons; that have the potential to crosstalk with Pdpn to influence neurogenesis; and which had been implicated in synaptic transmission, synaptic plasticity, neuronal morphology, neurogenesis and/or memory-related functions. Some of these proteins include RhoA (O’Kane et al., 2003, 2004; Dash et al., 2004; Wang et al., 2005; Martín-Villar et al., 2006; Diana et al., 2007), CDC42 (Okabe et al., 2003; Kim et al., 2014), Ezrin (Martín-Villar et al., 2006; Kim et al., 2010; Marsick et al., 2012), CD44 (Roszkowska et al., 2016), and NGF (Conner et al., 2009; Wang et al., 2012; Uzakov et al., 2015; Cicvaric et al., 2016). In particular, Ezrin is an interesting prospect to jointly mediate with Pdpn in the regulation of *in vivo* neurogenesis. In non-neuronal cells, it has been shown that Pdpn can exert its function *via* interactions with Ezrin, which is known to regulate neurotrophin-induced remodeling of F-actin and cell growth (Martín-Villar et al., 2006; Fernández-Muñoz et al., 2011; Smith and Melrose, 2011), and neuronal growth and morphology *via* redistribution of adhesion receptors (Sizemore et al., 2007). Recent studies have moreover unveiled Ezrin as a mediator of neurogenesis (Matsumoto et al., 2014) *via* down-regulation of the activity of RhoA, which is on itself a known downstream mediator of the Pdpn activity (Martín-Villar et al., 2006; Mahtab et al., 2009). Previous studies from our group have also demonstrated that brain neuronal Pdpn deletion results in altered levels of phospho-Ezrin upon stimulation with NGF (Cicvaric et al., 2016). Additionally, not only was NGF previously related to the regulation of neurogenesis and modulation of proliferation and migration of neural precursor cells (Maisonpierre et al., 1990; Chen et al., 2010; Birch et al., 2013; Tzeng et al., 2013; Zhang et al., 2013; Campos et al., 2014; Montalban et al., 2014; Oliveira et al., 2015; Sarma et al., 2015; Martorana et al., 2018; Mondal and Fatima, 2019), but our group has recently demonstrated (using Surface Plasmon Resonance) that Pdpn can directly interact with NGF and that Pdpn deletion alters the response of brain neurons to NGF stimulation (Cicvaric et al., 2016). Furthermore, both Pdpn and Ezrin protein expression have been identified in the neurogenic plexus choroids (Ming and Song, 2011) lateral ventricles region (Cleary et al., 2006; Kaji et al., 2010; Persson et al., 2010; Sathyanesan et al., 2012; Moon et al., 2013; Tomooka et al., 2013). Additionally, both Pdpn and the p75 NGF Receptor have been also previously associated with the regulation of cell migratory activity of NSCs and their invasive motility properties (Sailer et al., 2013). All these observations, together with our data here, provide several hints for future research aiming to elucidate the specific molecular pathways mechanistically linking Pdpn to *in vivo* neurogenesis in the adult mammalian brain.

### Pdpn Mediates in the Neuroprotective Effects of NGF

The experimental use of neurospheres cultures is widespread, as under specific conditions one can differentiate neurospheres into a variety of cells, including neurons, which provide thus a useful tool to examine molecular events critical for both the proliferation and differentiation stages of neurogenesis (Siebzehnrubl et al., 2011; Morte et al., 2013). In the *in vitro* experiments using neurospheres presented here, knockdown of Pdpn induced highly significant extensive cell death starting 4 days after cell differentiation (Figure 4A), indicating that Pdpn might be implicated in cell survival. Interestingly, this phenomenon was only observed in non-mitotic neuronal cells, as lack of Pdpn had no effect on the glial cell population (Figure 4B). NGF is a critical modulator of brain neuronal survival, neurotransmission, synaptic plasticity, and learning memory functions (Zhou et al., 1995; Conner et al., 2009; Zhang et al., 2013). NGF has been additionally implicated in the molecular signaling pathways regulating hippocampal neurogenesis (Birch et al., 2013; Zhang et al., 2013; Toyoda et al., 2014; Martorana et al., 2018). Our group has previously examined the involvement of Pdpn in neuritic outgrowth under basal conditions and in response to NGF using hippocampus-derived cultured neurons from Pdpn^+/+^ and Pdpn^−/−^ mice, and found that Pdpn mediates in the effects of NGF on hippocampal neuron neuritic outgrowth (Cicvaric et al., 2016). These observations support our hypothesis for the role of Pdpn in neuronal survival and for the Pdpn-dependent incorporation of newly generated neurons into existing circuits through cooperative signaling with NGF. To challenge this hypothesis, we cultured NSCs from wild type and Pdpn knockout mice for 4 days following differentiation and observed significantly higher amounts of cell death in cultures lacking Pdpn; an effect that was completely rescued by NGF treatment (Figure 4A). Given the importance of cell survival for the process of neurogenesis (Baptista and Andrade, 2018), our data thus for the first time propose Pdpn not only as a potential molecular candidate to influence neurogenesis, but also as a possible molecular element mediating in the NGF-dependent cellular mechanisms preserving the integrity of recently formed neurons *in vivo*.

### Pdpn: A Bidirectional Regulator of Synaptic Plasticity

During the last 40 years, a large number of studies on hippocampal synaptic plasticity have proven LTP as a powerful experimental tool to study the mechanisms that might lie beneath the hippocampal function, and learning acquisition and memory retention (Nguyen et al., 1994; Toni et al., 1999; Abraham and Williams, 2003; Sweatt, 2003a,b; Zakharenko et al., 2003). Information continues to be comparatively scarce, however, relating to molecular elements influencing hippocampal LTD (Kemp and Manahan-Vaughan, 2004; Etkin et al., 2006; Malleret et al., 2010). Previous reports have demonstrated that alterations in DG neurogenesis can influence both synaptic potentiation and synaptic depression at CA3-Schaffer collateral-CA1 and Medial Perforant Path-DG synapses (Wang et al., 2000; Snyder et al., 2001, 2005; Schmidt-Hieber et al., 2004; Saxe et al., 2006; Ge et al., 2007; Massa et al., 2011). These observations indicate that neurogenesis not only does not exclusively influences synaptic activity at a single hippocampal sub-region but its effects are not limited to synaptic potentiation. Indeed, in this type of experiment, the reestablishment of neurogenesis results in the full restitution of both synaptic potentiation and depression, with the latter requiring a much longer time to reach complete recovery (Massa et al., 2011). The molecular elements relating neurogenesis to both LTP and LTD remain, however, poorly characterized (Holderbach et al., 2007). Additionally, previous studies have established that both LTP and LTD are influenced by adult neurogenesis in the DG (Kitamura et al., 2010; Massa et al., 2011; Alam et al., 2018). It has been also shown that while newborn neurons—following their incorporation in the DG network—can improve the facilitation of the expression of LTP and LTD in DG, this process happens in a sequential time-dependent manner (Massa et al., 2011). In our previously established Pdpn knockout mouse model, we reported that the deletion of Pdpn selectively impairs long-term synaptic plasticity in the hippocampal DG (Cicvaric et al., 2016). However, the understanding of the contribution of Pdpn to synaptic transmission and plasticity in the DG remains far from complete. For example, while an impact of Pdpn deletion on synaptic potentiation was unveiled (Cicvaric et al., 2016), other forms of plasticity, such as synaptic depression, remained to be examined for its possible relation to the Pdpn function. Here, we expand these studies and further unveil that Pdpn gene deletion also influences LTD selectively at the DG (Figure 2). Our data thus for the first time propose Pdpn as one of the potential *in vivo* bidirectional and sub-region selective regulators of neurogenesis and memory-related synaptic plasticity.

### Podoplanin and the Crosstalk Between Neurogenesis, Synaptic Depression, and Anxiety-Like Behavior

Using the mouse as an experimental model organism, we here implemented for the first time a basic behavioral screening addressing the effects of Pdpn gene deletion on anxiety-related behavior. Previous research had established a physiological link between altered neurogenesis and depression- (Jacobs et al., 2000; Sahay and Hen, 2007; Lucassen et al., 2010; Eisch and Petrik, 2012) and anxiety-like behaviors (Revest et al., 2009; Baptista and Andrade, 2018). Our data had additionally shown that the deletion of Pdpn resulted in both altered neural progenitor cell proliferation and impaired LTD specifically at the neurogenic DG. LTD has been associated to memory-related cognitive impairment and to anxiety and mood-related disorders (Rolls, 2013; King et al., 2014; Leal et al., 2017). Changes in adult neurogenesis in the DG are also known to be importantly implicated in the anxiety- and depression-like behaviors in experimental animal models (Santarelli et al., 2003; Warner-Schmidt and Duman, 2006; Lagace et al., 2010; Parihar et al., 2011; Eisch and Petrik, 2012; Petrik et al., 2012; Aiello et al., 2015; Anacker et al., 2018; Cantacorps et al., 2018). Our experimental results indicated, for the first time, that Pdpn deletion associates with an apparent preference for avoiding the brightly lit center of an open space in the OF test (Figure 3A), which is a behavior indicative of increased anxiety (Olivier et al., 2008; Bailey and Crawley, 2009; Hughes et al., 2014; Kulesskaya and Voikar, 2014; Sturman et al., 2018). These observations were consistent with the data obtained during the EPM test (Figure 3C), which is also widely used as an indicator of anxiety-related behavior (Bailey and Crawley, 2009) and which further propose the possibility that Pdpn might serve a physiological role in the prevention of the expression of anxiety-related behaviors that might occur in concomitance with altered neurogenesis. Only the LDB test showed no significant differences between wild type and Pdpn knockout animals (Figure 3B). Differences in results in the LDB could be therefore indicative of subtle variances in the manifestation of what is defined as “mood,” which in certain cases might not be reflected in all of the different tests. For example, other groups have described that differences in the results from behavioral mood-related tests might become apparent not only due to the intrinsic differences of the experimental setting itself (as for example possible changes in the transparency or the degree of illumination) but can become exacerbated in specific strains, pharmacological interventions or genetic modifications (Hogg, 1996; Bourin and Hascoét, 2003; Hagenbuch et al., 2006; Ramos, 2008; Violle et al., 2009; Miller et al., 2011; Steimer, 2011). Further research from other groups and using other behavioral tests (e.g., Social Interaction Tests), is therefore encouraged in order to independently validate our observations. Taken together, data presented here for the first time propose Pdpn as a potential molecular component mediating in the regulation of neurogenesis and in the expression of anxiety-related behaviors. Further experiments using alternative Pdpn-down-regulation approaches like CRISPR-Cas9-based (Cong et al., 2013; Horvath et al., 2017; Ma et al., 2017) targeted Pdpn gene deletion could additionally contribute to the verification of the here proposed *in vivo* role of Pdpn in neurogenesis, NGF-mediated neuroprotection and anxiety-related behaviors.

## Data Availability Statement

The datasets generated for this study are available on request to the corresponding author.

## Ethics Statement

The animal study was reviewed and approved by the National Ethical Committee on animal care and use (BMWFW104 66.009/0201-WF/II/3b/2014, Bundesministerium für Wissenschaft, Forschung und Wirtschaft).

## Author Contributions

FM conceived the project. AC and FM designed the experiments and performed most of the behavioral, electrophysiological, cell culture experiments and immunohistochemical analyses, supervised the data analysis, interpretation and jointly wrote the full body of the manuscript. HS helped with immunohistological studies and independently replicated parts of the immunohistological results. TSt performed immunocytological experiments and data quantification. TSm advised on molecular experiments and conducted data analysis. DS and TM independently replicated some of the cell culture experiments and helped with parts of the writing of the manuscript. PU provided the animals used in the study and helped with the data interpretation. All authors discussed the findings presented in this study and commented on the manuscript.

## Conflict of Interest

The authors declare that the research was conducted in the absence of any commercial or financial relationships that could be construed as a potential conflict of interest.
